# Ion Pair-HPLC Method for the Simultaneous Estimation of Quinapril and Hydrochlorothiazide in Tablets

**DOI:** 10.4103/0250-474X.56035

**Published:** 2009

**Authors:** M. Gandhimathi, T. K. Ravi

**Affiliations:** Department of Pharmaceutical Analysis, College of Pharmacy, Sri Ramakrishna Institute of Paramedical Sciences, 395-Sarojini Naidu Road, New Siddhapudur, Coimbatore-641 044, India

**Keywords:** Quinapril, hydrochlorothiazide, ion pair-HPLC, validation

## Abstract

A simple, precise and rapid HPLC method has been developed and validated for the estimation of quinapril and hydrochlorothiazide simultaneously in combined dosage form. The mobile phase used was a mixture of 0.1% v/v triethylamine (pH 3.5), containing 1 mM of hexane sulphonic acid: acetonitrile (30:70% v/v). The detection of quinapril and hydrochlorothiazide was carried out on photo diode array detector at 220 nm. Results of the analysis were validated statistically and by recovery studies. The proposed method can be successfully used to determine the drug contents of marketed formulation.

Hydrochlorothiazide (HZ)[1] is a benzothiazidine derivative used as diuretic agent. Quinapril[1] (QP) is an isoquinoline carboxylic acid derivative used in treatment of hypertension. Literature survey[2–11] revealed that only a few analytical methods including LC-MS, HPLC methods were reported for the determination of quinapril and its metabolite, in dosage form and plasma. HPLC and LC-MS methods have been reported for the estimation of hydrochlorothiazide alone and also in combination with other drugs. A derivative spectroscopy and chemometric methods have been reported for the simultaneous estimation of quinapril and hydrochlorothiazide. However, so far no HPLC method has been reported for simultaneous estimation of quinapril and hydrochlorothiazide in combination. Hence, this paper reports a simple, precise and accurate HPLC method for the simultaneous estimation of quinapril and hydrochlorothiazide from combined dosage form.

Separation was carried out on an isocratic HPLC system, Shimadzu LC-Class 10AT VP pump with PDA detector, Class LC 10 software and RP-C_18_ Gemini (150×4.5 mm, 5 μ). The chromatographic estimation was performed using the following conditions: the mobile phase used was of 0.1% v/v triethylamine (pH 3.5), containing 1 mM of hexane sulphonic acid: acetonitrile (30:70 %v/v). The run time and flow rate were 6 min and 1 ml/min, respectively. Detection wavelength was set at 220 nm.

Standard stock solutions 1 mg/ml of QP and 1.2 mg/ml of HZ were prepared in acetonitrile. For construction of calibration graph, working standard solution were prepared by further diluting the stock solution with mobile phase to get a concentration of 30-150 μg/ml of QP and 40-200 μg/ml of HZ. This method was applied to determine QP and HZ in combined formulation of market samples. For the analysis of tablet formulation an accurately weighed tablet powder equivalent to 10 mg of QP and 12.5 mg of HZ were taken in a volumetric flask. The powder was dissolved in acetonitrile, shaken thoroughly and made upto the volume with acetonitrile, Then the solution was filtered through Whatman filter paper No. 41 and further diluted with mobile phase to get the concentration of 90 μg/ml and 120 μg/ml of QP and HZ, respectively. These solutions were injected and the chromatograms were recorded. A typical chromatogram of HZ and QP is shown in fig 1.

**Fig 1:**
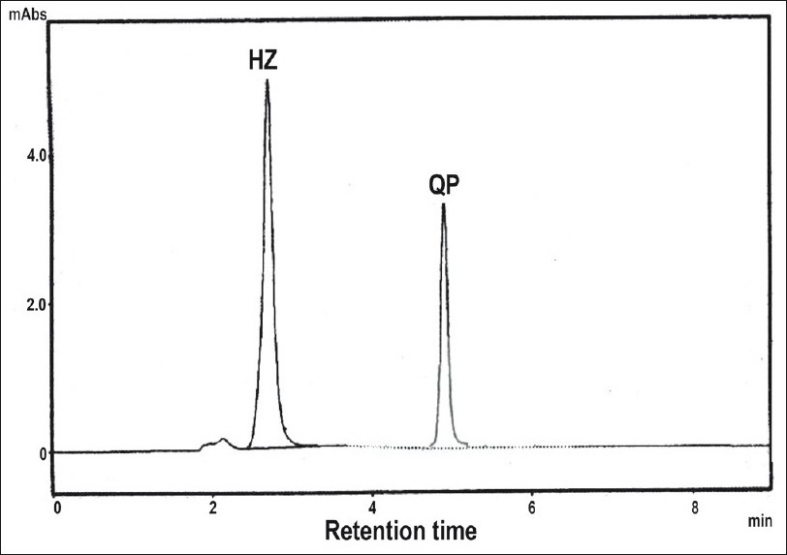
A typical chromatogram of HZ and QP. A typical chromatogram of hydrochlorothiazide (HZ) and quinapril (QP)

The method was validated in terms of linearity, accuracy, inter-day and intra-day precision, reproducibility and specificity. The limit of detection and limit of quantification was also determined. The accuracy of the method was evaluated by carrying out recovery studies. For that, known concentrations of standard were added to the pre-analyzed sample solution and the recovery was calculated. The precision was determined by analyzing standard solutions in the linearity range of calibration curve in triplicate on the same day (intra day precision) and different days (inter day precision). The corresponding relative standard deviation were determined and found to be < 2.5. The validated data is furnished in Table 1.

**TABLE 1:** SYSTEM SUITABILITY TEST PARAMETERS FOR QUINAPRIL AND HYDROCHLOROTHIAZIDE BY PRPPOSED METHOD

System suitability	Results	
	
Parameters	Quinapril	Hydrochlorothiazide
Theoretical plates	8837	6551
Resolution	-	3.31
Linearity (μg/ml)	30-150	40-200
Correlation coefficient	0.9946	0.9953
% Recovery	98.78	99.38
LOD (μg/ml)	0.05	0.02
LOQ (μg/ml)	0.4	0.1
Tailing factor	1.13	1.09

LOD: Limit of detection and LOQ: limit of quantitation

Both QP and HZ are soluble in acetonitrile, therefore it was selected as solvent. The mixture of 0.1% v/v triethylamine (pH 3.5), containing 1 mM of hexane sulphonic acid:acetonitrile (30:70% v/v) could resolve QP and HZ with better resolution. The retention times of QP and HZ are 4.5 min and 1.8 min, respectively. Linearity range for QP and HZ were 30-150 μg/ml (r = 0.9946) and 40-200 μg/ml (r = 0.9953), respectively. The linear regression equations are Y= 348 +52.9X for QP and Y= 19.299+173.209X for HZ.

The high percentage of recovery of the drugs indicates that the method is highly accurate and reliable (Table 2). The content and the percentage of drugs in market samples (Table 3) indicate that the proposed method is simple, rapid, precise and accurate for the estimation of quinapril and hydrochlorothiazide in its pharmaceutical formulation.

**TABLE 2:** RECOVERY STUDY OF PROPOSED METHOD

Drug	Amount of standard added (mg)	Amount recovered (mg)	% Recovery
Quinapril	5	5.11	102.20
	10	10.25	102.50
Hydrochlorothiazide	6	5.98	99.67
	12	11.96	99.66

**TABLE 3:** RESULTS OF ANALYSIS FOR QUINAPRIL AND HYDROCHLORORTHIAZIDE IN FORMULATION

Drug	Amount (mg/tablet)		%Label claim*	%RSD*
	
	Labeled	Found*		
Quinapril	20	19.94	99.70	1.3733
Hydrochlorothiazide	25	24.97	99.89	0.7767

An average value±relative standard deviation of 6 observations
